# TailBoost: Tail-Synthetic Learning for Boosting Long-Tailed Skin Cancer Image Classification

**DOI:** 10.3390/s26113343

**Published:** 2026-05-25

**Authors:** Tianyunxi Wei, Yijin Huang, Li Lin, Pujin Cheng, Xiaoying Tang

**Affiliations:** 1 Department of Electronic and Electrical Engineering, Southern University of Science and Technology, Shenzhen 518055, China; 11911238@mail.sustech.edu.cn (T.W.); yijinh@student.ubc.ca (Y.H.); linlimir@connect.hku.hk (L.L.); 2 School of Biomedical Engineering, The University of British Columbia, Vancouver, BC V6T 1Z4, Canada; 3 Department of Electrical and Electronic Engineering, The University of Hong Kong, Hong Kong 999077, China; chengpj@connect.hku.hk; 4 Jiaxing Research Institute, Southern University of Science and Technology, Jiaxing 314000, China

**Keywords:** long-tailed learning, skin cancer recognition, saliency map, mixup, supervised contrastive learning

## Abstract

Skin cancer image data often exhibit long-tailed distributions due to the inherent challenges in data collection and annotation. Specifically, a few predominant classes dominate a dataset of interest, while minority classes, referred to as tail classes, are underrepresented with only limited numbers of samples. Such imbalance is highly likely to adversely affect the performance of deep learning models. To address this issue, previous methods employ mixup techniques to synthesize tail-class images, thereby attempting to balance the training data. However, traditional mixup methods typically do not specifically pay attention to specific regions of interest, blending two images with indistinction between objects of interest and background. Such disregard for important semantic features may result in synthetic samples with broken or distorted diagnostic features. In this work, we introduce a novel framework, the Tail-synthetic Learning for Boosting Long-tailed Skin Cancer Image Classification (TailBoost) framework. Our approach generates a new tail-class image by combining a tail-class image with a head-class image under the guidance of their corresponding saliency maps. This strategy, namely SPMix, preserves and enhances the discriminative features of the tail-class image with minimum interference from the head-class image. We further refine the learned representations by incorporating supervised contrastive learning with class-center rebalance. Extensive experiments on the ISIC2018, ISIC2019, and PAD-UFES-20 datasets demonstrate that TailBoost outperforms existing state-of-the-art long-tailed learning methods.

## 1. Introduction

Skin cancer is a common type of cancer characterized by abnormal proliferations of skin cells, primarily caused by prolonged exposures to ultraviolet (UV) radiation from the sun or tanning beds. The main types of skin cancer include basal cell carcinoma (BCC), squamous cell carcinoma (SCC), and melanoma [1]. Early detection and preventive measures, such as regular skin examinations and sun protection, are crucial for effectively reducing the risk of skin cancer. Accurate diagnosis of skin cancer is of utmost importance. Traditional diagnostic methods, such as visual inspection and biopsy, can be time-consuming, subjective, and prone to human error. In recent years, deep learning techniques have shown significant promise in enabling automated diagnosis of skin cancer utilizing dermoscopic images.

Over the past decade, deep learning has led to significant advancements in visual recognition, with notable breakthroughs in classification tasks [2,3]. In traditional classification settings [4,5], training data are typically balanced to ensure almost equal sample sizes across different classes. However, medical datasets, especially those related to skin cancer, often exhibit long-tailed distributions. As discussed in recent surveys [6], long-tailed datasets differ from generic imbalanced datasets in that the test set is commonly class-balanced, such that all classes are evaluated equally despite the imbalance presented in the training data. In this scenario, head classes associated with common conditions dominate the dataset, while tail classes corresponding to rare but clinically important conditions remain underrepresented. This imbalance arises due to challenges in acquiring data for rare diseases and the high costs involved in annotating medical images. Training deep learning models on such imbalanced datasets presents significant challenges, as low-frequency tail classes risk being overshadowed by the dominant head classes, leading to reduced performance on these underrepresented classes. Long-tailed skin cancer image classification is especially crucial because real-world clinical datasets exhibit severe class imbalance. Common lesion types overwhelm the dataset, while rarer yet clinically critical lesions, such as specific melanoma subtypes, are significantly underrepresented. This imbalance increases the risk of misdiagnosis for rare lesions, emphasizing the need for improved methods to recognize these tail classes. The importance of this work lies in addressing the challenges posed by long-tailed distributions in skin cancer image classification and ultimately enhancing the accuracy and reliability of diagnosing both common and rare lesions.

**Figure 1:**
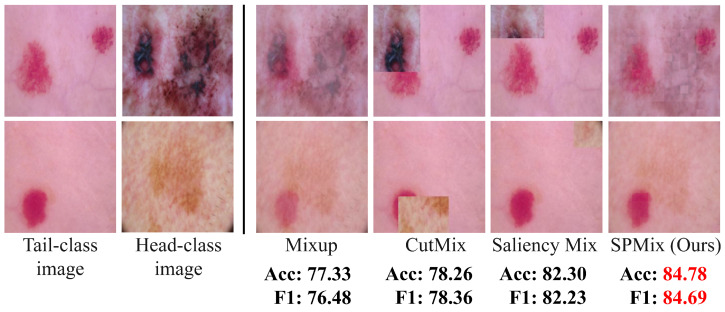
A comparison of different mixup methods applied to a tail-class image and a head-class image. The accuracy and F1-score of our proposed TailBoost with different mixup strategies on the ISIC 2018 dataset are presented.

To address the challenge of imbalanced data, class re-balancing or re-weighting [7,8,9] are proposed to rebalance the original data distribution. These approaches, however, either increase the risk of overfitting to tail classes or degrade the performance of head classes. Data augmentation is also a widely adopted technique to mitigate data imbalance by synthesizing additional samples for tail classes, offering a cost-effective solution to the scarcity of tail-class samples. In this context, the Mixup method [10] is often employed, which works by blending two images together using interpolation techniques to synthesize new tail-class samples, thereby balancing the dataset of interest and improving the representation of tail classes during training. CutMix [11] has also gained significant attention due to its ability to enhance the diversity of tail-class samples by replacing a random region in an image with a patch from another image, along with combining their labels proportionally. SaliencyMix [12] improves upon CutMix by leveraging salient information to identify the most prominent region, which is then inserted into a target image at the corresponding position. Although these methods have demonstrated success in enhancing tail-class representation in natural image datasets, their effectiveness is limited when applied to dermoscopic images. They tend to overlook the intrinsic semantic information in tail-class images, which is crucial for accurate classification. For example, SaliencyMix selects a randomly sized patch surrounding the most salient pixel. However, lesions in dermoscopic images may be distributed throughout the entire image, so the patch selected in SaliencyMix may not cover all lesion areas and thus cannot accurately represent the key diagnostic features of the class. Dermoscopic images often contain lesions with fuzzy and irregular boundaries, delicate pigment networks, and subtle local structures such as streaks, dots, and globules—patterns that are diagnostically crucial yet sensitive to spatial distortion. Traditional methods like Mixup and CutMix, which randomly combine images or linearly interpolate pixel values, fail to preserve the semantic integrity of these structures. The naive pixel-level mixing process often distorts fine-grained lesion details, blending lesion edges with unrelated background textures or introducing features from head-class lesions into tail-class samples, as visually demonstrated in Figure 1. This disrupts the accurate representation of tail classes and can produce physiologically implausible mixed images, thereby diminishing the effectiveness of model training. This may lead to inconsistency between the synthetic image’s label and its semantic information, thereby diminishing the effectiveness of model training. Such label-versus-semantics inconsistency is a common issue to most mixup methods. Additionally, these methods rely on predefined or random mixing ratios that do not adapt to the varying importance of different regions within an image. Such lack of adaptability may result in either overemphasis or underrepresentation of lesion regions, diminishing the model’s ability to capture essential features that distinguish different classes. This limitation can hinder the learning of representations for tail classes, since not considering semantic relevance may generate synthetic samples that lack the necessary details to accurately represent tail classes. It is particularly problematic for dermoscopic images, where accurate diagnosis depends on the identification of critical lesion information. To sum up, these methods are vulnerable to compromising the integrity of synthetic images by either obscuring lesion details or producing labels that do not align with the actual semantic content of the synthetic images.

In this work, we propose a contrastive framework with an enhanced mixup strategy for long-tailed skin cancer image classification, namely TailBoost. Specifically, we employ saliency maps to guide patch-based, feature-wise mixup (SPMix). A saliency map is utilized for distinctly highlighting salient lesion regions from the surrounding background. Our objective is to synthesize new samples that preserve diagnostic structures within tail classes while enriching background diversity using head-class samples.

**Figure 2:**
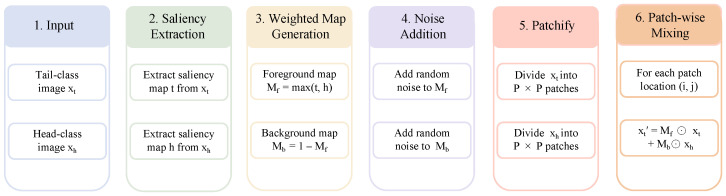
Detailed schematic flowchart of the proposed SPMix pipeline.

To improve the clarity of the proposed pipeline, the overall procedure of SPMix is illustrated in Figure 2. The framework first extracts saliency maps from head-class and tail-class images, followed by saliency normalization and thresholding to stabilize foreground localization. A lightweight random perturbation term is then introduced to increase augmentation diversity. Afterward, images are divided into patch tokens through a patchify operation, and patch-level saliency scores are used to generate adaptive mixing weights for feature-wise synthesis.

Unlike conventional Mixup methods that directly interpolate pixels, SPMix performs localized mixing at the feature level, preserving lesion structure and semantic consistency while remaining compatible with Transformer-based architectures. We further design a supervised contrastive learning (SCL) framework based on momentum contrast [13] to enhance representation learning under long-tailed distributions.

TailBoost integrates SPMix, patch-based feature-level augmentation, and supervised contrastive learning with class-center rebalancing. SPMix uses saliency-guided mixup to generate samples that emphasize clinically relevant regions and preserve tail-class features. Patch-level augmentation operates in the feature space, refining the fusion process. Contrastive learning with class-center rebalancing enhances intra-class compactness and inter-class separability, especially for tail classes. These components work together to improve tail-class feature learning and mitigate the effects of long-tailed distributions. The source code is available at https://github.com/Yancy10-1/SPMix (accessed on 17 May 2026). In summary, our main contributions are as follows:

- We propose a novel method, TailBoost, for long-tailed skin cancer classification. TailBoost comprises two components: SPMix and a supervised contrastive learning framework. SPMix synthesizes tail-class samples to balance the training data, while the SCL framework is designed for better tail-class representation learning.
- We introduce SPMix as a lesion-aware mixup strategy, leveraging saliency maps to synthesize tail-class images that preserve key diagnostic features, thereby providing semantically consistent labels and improving the representation learning power for tail classes.
- We validate SPMix as a plug-and-play module that can be integrated into various long-tailed learning frameworks, leading to consistent performance improvements.
- Extensive experiments are conducted on three datasets, demonstrating the effectiveness of TailBoost for long-tailed skin cancer classification.

This paper extends our SPMix [14] in several aspects:

- We simplify the model architecture by eliminating the need for a separate momentum encoder.
- We investigate the applicability and flexibility of SPMix, demonstrating its effectiveness and adaptability as a plug-and-play module. Our results show that incorporating SPMix significantly improves the performance of various long-tailed learning methods.
- We conduct extensive experiments on two additional long-tailed skin cancer datasets and compare with more state-of-the-art methods, demonstrating the superiority of our proposed framework.
- We perform ablation studies on the ISIC 2018 dataset to more thoroughly analyze the impact of each module.
- We visualize representative synthetic tail-class images to demonstrate how SPMix preserves the diagnostic features of tail classes while avoiding head-class’s interference.

## 2. Related Work

### 2.1. Long-Tailed Learning

#### 2.1.1. Class Rebalancing-Based

Medical data often exhibit long-tailed or imbalanced distributions, which bias the learning process toward head classes and lead to degraded performance on tail classes. Resampling methods attempt to mitigate this problem by adjusting the training distribution through undersampling or oversampling. Undersampling approaches [15,16], which discard a large portion of majority-class samples, may result in the loss of valuable information and are often impractical under severe imbalance. Oversampling techniques [17] increase the number of tail-class instances by replicating existing samples; while simple and broadly applicable, they risk overfitting since duplicated samples do not introduce new variations. Re-weighting strategies adjust sample importance either at the class level or instance level [18,19], or modify logits directly [20] to boost tail-class performance. Common class-level weighting schemes, such as inverse-frequency weighting [21], attempt to compensate for skewed data distributions. LDAM [22] further improves tail-class learning by introducing a margin proportional to class frequency, eliminating the need for reweighting at the early training stage and enabling more effective feature learning for minority classes. Several studies have also explored how reweighting can guide classifier learning. For instance, Kang et al. [23] propose a decoupled training framework in which feature representations are first learned using uniform sampling, followed by classifier fine-tuning with class-balanced sampling, while Zhou et al. [24] introduce a cumulative learning strategy that first captures universal patterns and then gradually focuses on tail classes.

Beyond sample- and loss-level adjustments, recent class-rebalancing approaches such as Taming the Tail [25], which employs an asymmetric Padé-approximated loss for medical long-tailed data, and MORE [26], which rebalances model parameters via low-rank decomposition and sinusoidal reweighting, highlight the importance of optimization- and parameter-level correction. Complementing these efforts, recent analyses of long-tailed representation geometry [26] reveal that tail-class feature centers tend to shrink and overlap in the feature space under severe imbalance, making them harder to discriminate. These findings underscore that effective long-tailed learning must also address representation-level imbalance, motivating the development of methods that explicitly reshape or regularize the feature space to preserve discriminative tail-class information.

#### 2.1.2. Contrastive Learning-Based

Recently, contrastive learning has been widely explored for long-tailed recognition. Self-supervised approaches [13,27,28] learn discriminative representations by maximizing similarity between positive pairs while separating negative pairs. Supervised Contrastive Learning (SCL) [29] further incorporates label information to improve class-aware representation learning. However, under long-tailed distributions, contrastive learning may still become biased toward head classes due to severe imbalance in positive and negative samples.

Several methods have attempted to alleviate this issue. Hybrid-SC [30] combines supervised contrastive learning with a balanced classifier, while KCL [31] reduces sampling imbalance through class-balanced positive pair construction. PaCo [32], BCL [33], and ProCo [34] further improve long-tailed representation learning by introducing class centers, balanced contrastive objectives, or class-distribution modeling. More recent studies such as ACL [35] and BCE3S [36] continue to enhance representation balance through improved optimization strategies.

Different from previous methods, the proposed TailBoost framework combines supervised contrastive learning with saliency-aware patch-level feature fusion and learnable class centers. This design preserves lesion-related semantic structures during augmentation while explicitly enhancing tail-class feature aggregation and representation balance, making it more suitable for long-tailed medical image classification.

### 2.2. Data Augmentation

In recent years, data augmentation techniques have been widely adopted to enrich dataset diversity by synthesizing new samples. Early approaches such as Cutout [37] remove random image regions, while Mixup [10] performs linear interpolation between pairs of training images. CutMix [11], which combines the strengths of both, replaces a removed region of an image with a patch from another image and assigns a mixed label proportional to the replaced area. However, these traditional augmentation methods rely on random spatial selection or pixel-level interpolation and therefore overlook the semantic structure of medical images. In the context of skin lesion classification, such naive image-level mixing can unintentionally introduce background or head-class lesion information into tail-class samples, weakening the already subtle and scarce semantic cues of rare lesions and ultimately degrading performance.

To alleviate this, SaliencyMix [12] utilizes saliency maps to identify visually important pixels and restricts patch selection to foreground regions. Although this approach reduces the incorporation of background noise, its pixel-level saliency-guided cropping and pasting in the image space can still produce boundary artifacts, texture inconsistencies, and semantic discontinuities, which are particularly harmful in medical images where lesion morphology must remain coherent. ConCutMix [38] further improves regional replacement by introducing consistency regularization during CutMix augmentation. However, its image-level region substitution strategy may still introduce semantic interference between lesion and background regions, especially in fine-grained skin lesion recognition tasks. DiffuseMix [39] adopts diffusion-based image synthesis to improve sample diversity for long-tailed learning, but it relies on iterative denoising procedures and auxiliary generative models, leading to increased computational overhead and training complexity. Moreover, the quality of generated samples is highly dependent on diffusion generation stability. Recent studies have reported that diffusion-generated medical images may introduce unrealistic textures, semantic inconsistencies, or distorted pathological structures, particularly for minority classes with limited training samples [40,41]. Such issues may prevent the synthesized images from accurately reflecting the true variability of tail-class lesions and may weaken clinically important diagnostic characteristics. In contrast, our method directly enhances tail-class representations through saliency-guided feature interaction without requiring additional generative sampling, thereby avoiding the instability and semantic distortion risks introduced by diffusion-based synthesis.

In contrast, our method performs saliency-guided feature interaction directly in the representation space through patch-level semantic mixing. Instead of replacing image regions in the pixel space, the proposed SPMix maps saliency information into semantically aligned feature patches and adaptively enhances tail-class lesion representations while preserving structural continuity. This design effectively avoids pixel-level artifacts and maintains lesion morphology consistency. Furthermore, unlike DiffuseMix, our framework does not require additional generative sampling or iterative diffusion procedures, resulting in lower computational complexity and more stable optimization. Compared with ConCutMix, our method additionally incorporates class-aware representation refinement and supervised contrastive optimization to explicitly improve minority-class feature separability and representation balance under long-tailed distributions. These properties make the proposed framework more suitable for long-tailed medical image classification scenarios where semantic consistency and structural preservation are critical.

MiSLAS [42] uses mixup during the initial training phase to improve the performance of tail classes through resampling. Remix [43] assigns labels to multiple classes when combining two samples. ConCutMix [38] generates augmented samples to improve recognition in long-tailed distributions. It calculates similarities across samples in the semantic space of contrastive learning and adjusts the region-based labels accordingly. Our approach samples images from different distributions and mixes them based on the images’ saliency, providing labels that are semantically consistent with tail classes and overcoming the limitations imposed by long-tailed distributions.

## 3. Methodology

**Figure 3:**
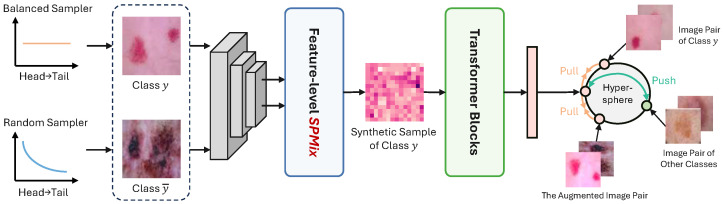
The overall pipeline of our TailBoost framework involves several key steps. We begin by performing SPMix upon images sampled from both a balanced sampler and a random sampler to generate synthetic samples. Features from these images are extracted using a same CNN. After feature-level SPMix, the mixed features are input to transformer blocks, and the resulting features are used to compute supervised contrastive loss. We employ supervised contrastive learning to create a semantic space in which TailBoost minimizes distances between positive samples with similar features and maximizes distances between negative samples from different classes.

The overall framework of our proposed TailBoost is illustrated in Figure 3, which comprises three key components: (1) Synthetic images are generated by mixing tail-class and head-class images to create a balanced training dataset. (2) Features are extracted and feature patches are mixed under the guidance of saliency maps. (3) The mixed features are further encoded using transformer blocks, and SCL is employed for representation learning.

### 3.1. Vision Transformer

In this section, we briefly introduce the mechanism of vision transformer (ViT) [44]. The core idea behind ViT is to divide images into smaller patches and treat each patch as an element in a sequence processed by transformer blocks [45]. An image $x\in R^{H\times W\times C}$ is reshaped into a sequence of flatten 2D patches $x_{p}\in R^{N\times P^{2}\times C}$, where (H,W) is the original image resolution. *C* is the number of channels, (P,P) is the resolution of each image patch, and the resulting image patch number $N=H\times W/P^{2}$, which corresponds to the effective input sequence length of the ViT. These patches are then projected into a space of dimension *D* by a learnable linear projection layer, resulting in patch embeddings $x_{P}\in R^{N\times D}$. The position embeddings $E_{pos}\in R^{N\times D}$ are added to the patch embeddings to retain spatial position information. The input is then processed by multiple transformer blocks, each consisting of a multi-headed self-attention (MSA) layer and a multi-layer perceptron (MLP) block.

The transformer layer helps the model capture relationships between different patches of the image, enabling it to identify global features across the entire image rather than focusing only on local regions. CNNs, on the other hand, are more effective at detecting local patterns because they primarily rely on convolutional kernels. To combine the advantages of both approaches, hybrid architectures, such as Hybrid-ViT [46], are proposed. In our framework, we adopt this architecture to leverage the local feature extraction of CNNs and the global feature identification of ViTs. Specifically, our framework employs the ResNet-50 architecture to generate feature maps, which are then input into the transformer blocks of ViT-S, a variant of the ViT model.

### 3.2. Saliency-Guided Mixup

To address the issue of long-tailed distributions, where models tend to focus disproportionately on dominant (head) classes due to class imbalance, we adopt a mixup-based strategy to synthesize tail-class samples and mitigate the scarcity of tail-class data. However, traditional mixup methods generally fail to focus on key regions of interest, blending two images without distinguishing between the objects of interest and the background. This may result in synthetic samples that lack important diagnostic features or generate samples with inconsistent labels, leading to misleading training signals. For long-tailed skin cancer classification, the key to providing samples consistent with their labels lies in preserving the lesion information within tail-class samples while enriching the diversity of their image backgrounds. To achieve this, we employ saliency map to guide the mixup strategy. Saliency map highlights the most conspicuous regions in an image—in this case, the lesions—where pixel values indicate the degree of saliency [47]. We employ a static saliency detection method [48] that evaluates saliency by computing central-surround differences within the image of interest. This approach is particularly effective for dermoscopic images, which often exhibit distinct intensity contrasts between lesion regions and their surrounding backgrounds. In our framework, the static saliency detection module is implemented using cv2.saliency.StaticSaliencyFineGrained [49], a well-established fine-grained visual saliency model. Prior to saliency extraction, a lightweight preprocessing pipeline is applied to suppress common dermoscopic artifacts such as hair and imaging noise. Specifically, Gaussian filtering is first utilized to smooth the input image and remove fine-grained noise patterns. Morphological erosion and dilation operations are subsequently performed to refine lesion structures and improve foreground continuity. These preprocessing steps reduce the risk of artifacts being incorrectly highlighted as salient foreground regions during saliency estimation.

**Figure 4:**
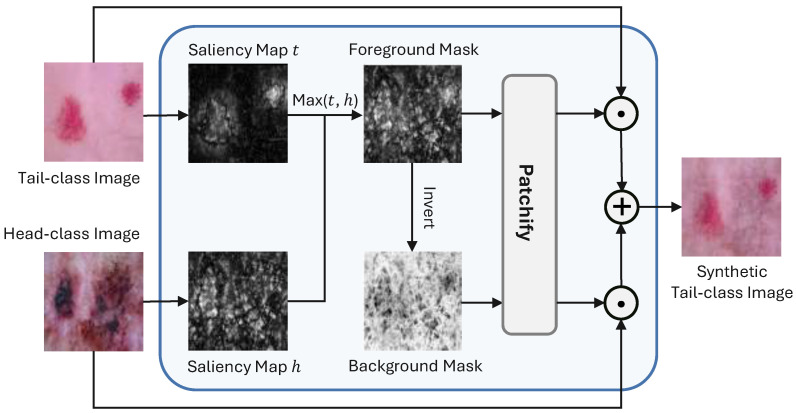
Illustration of our crucial module SPMix. The saliency maps of a head-class image and a tail-class image are operated to obtain a foreground mask and a background mask. The pixel values of the two masks are used as the mixing ratios. The scaling and noise addition operations are omitted from the figure for simplicity.

To guide the mixup process, as shown in Figure 4, we define two key components: the foreground mask and the background mask. The foreground mask is applied to a tail-class image, preserving its diagnostic features by focusing on the key lesion areas. In contrast, the background mask is applied to a head-class image to extract diverse background elements. Using both balanced and random samplers in SPMix stems from the need to address the imbalance between foreground lesion preservation and background diversity in long-tailed medical image classification. Specifically, the balanced sampler is employed to ensure the selection of tail-class images, from which the foreground mask is extracted to capture and preserve diagnostically significant lesion regions. This helps retain crucial class-specific features that are often underrepresented. On the other hand, the random sampler targets head-class images, providing a rich and diverse pool of background content. By extracting the background mask from these images, SPMix enhances context diversity without overwhelming the lesion-focused signal. This dual-sampling strategy promotes a more balanced feature learning process by ensuring that rare class-specific information is preserved while maintaining background variation to improve generalization. By combining these two masks, we generate a new tail-class image that retains the critical diagnostic features of the original tail-class image while incorporating diverse backgrounds from the head-class image. Specifically, we define the foreground mask as regions with higher saliency scores in both tail-class and head-class samples, while the complement of the foreground mask forms the background mask. This strategy ensures that salient regions unique to the head-class image are excluded from the synthetic tail-class image, while preserving the diagnostic saliency of the tail-class image to the greatest possible extent. Since the saliency map is static, merging two saliency map images always yields the same result, which could increase the risk of overfitting by generating repetitive synthetic images. We thus introduce a random perturbation term denoted as ϵ into the foreground masks to increase the diversity of synthetic samples and avoid deterministic mixing patterns during training. Specifically, the noise term is sampled from a zero-mean Gaussian distribution:

(1) $$\epsilon ∼N(0,\sigma^{2}),$$

where σ denotes the standard deviation controlling the perturbation magnitude. In addition, min-max normalization is applied to the saliency maps to scale the saliency scores into the range [0, 1]. However, some salient patches may have scores as high as 1, which results in mixing ratios of 1. This means these patches are not mixed, leading to less effective augmentation. To enable effective blending, we introduce a threshold, denoted as α, and cap scores exceeding this threshold at α. This approach ensures that all feature patches are included in the mixing process. Ultimately, we determine the mixing ratio, denoted as *m*, based on their respective positions. For a given tail-class sample $x^{t}$ and a head-class sample $x^{h}$, data augmentation generates two augmented views for each, with foreground and background masks calculated as follows:

(2) $$\begin{array}{cc} \; & m^{f}=\min\left(\alpha ,\max\left(s^{h},s^{t}\right)+\epsilon \right),\\ \; & m^{b}=1-m^{f}, \end{array}$$

where $s^{h}$ and $s^{t}$ denote the saliency scores of the head-class image, tail-class image respectively and $m^{f}$, $m^{b}$ represent the foreground mask and the background mask respectively. We then compute the saliency-guided mixing ratio $m^{f}$ and proceed to synthesize a new tail-class sample by

(3) $$\begin{array}{c} x^{t^{\prime }}=m^{f}⊙x^{t}+m^{b}⊙x^{h}, \end{array}$$

where $x^{t^{\prime }}$ is considered as a synthetic tail-class sample. ⊙ is element-wise product.

### 3.3. Patch-Based Mixup

Although saliency maps can help identify lesion regions, directly using their pixel values as the mixing ratios at the pixel level can lead to discontinuous lesion features. This is because saliency maps are sharp and may contain noise or abrupt changes in pixel values, which can cause the lesion area to appear fragmented or disconnected, disrupting the continuity of the lesion region in the synthetic image. This inconsistency in the lesion regions compromises feature coherence and potentially hinders model training. Given that ViTs process images by dividing them into smaller patches and treating these patches as sequence elements, we propose a feature-level, patch-based mixup approach to synthesize new images, which is naturally well-suited for ViTs. The feature-level, patch-based mixup is more effective at preserving lesion information in tail-class samples because it operates on higher-level features rather than individual pixels. This approach ensures that the critical lesion information remains intact, avoiding pixel-level artifacts that could break up or distort the lesion area, which is essential for maintaining the region’s diagnostic coherence.

We first divide the saliency map into non-overlapping patches and assign the average saliency score of each patch as its mixing ratio. Given that feature-level mixup is sensitive to the mixing coefficient, the patch-wise saliency score is further normalized to stabilize feature interaction. Applying patch-based mixup, the foreground mask from Equation (2) becomes:

(4) $$\begin{array}{cc} \; & {\bar{s}}_{ij}=\frac{1}{p^{2}}\sum_{u=0}^{p-1}\sum_{v=0}^{p-1}s_{i+u,j+v},\\ \; & m_{ij}^{f}=\min\left(\alpha ,\max\left({\bar{s}}_{ij}^{h},{\bar{s}}_{ij}^{t}\right)+\epsilon \right),\\ \; & m_{ij}^{f}=m_{ij}^{f}/2+0.5. \end{array}$$

Here, $s_{i+u,j+v}$ denotes the saliency value at spatial position (i+u,j+v), and *p* represents the patch size. ${\bar{s}}_{ij}$ is the average saliency score of the (i,j)-th patch, reflecting the importance of the local region. ${\bar{s}}_{ij}^{h}$ and ${\bar{s}}_{ij}^{t}$ denote the patch-wise saliency scores of the head-class and tail-class samples, respectively. The operator max(·) selects the more salient response between the two samples to preserve lesion-related regions during mixing. ϵ represents Gaussian random noise sampled from $N(0,\sigma^{2})$, which introduces stochastic perturbation to improve sample diversity and avoid repetitive mixing patterns. α is an upper-bound threshold used to prevent overly dominant saliency weights. The final normalization step rescales the foreground mask into the range [0.5, 1], ensuring that tail-class lesion features maintain a dominant contribution during the feature fusion process. This design encourages the synthesized representations to preserve diagnostically relevant minority-class characteristics while still incorporating complementary contextual information from head-class samples. Empirically, setting the lower bound to 0.5 provides a stable balance between semantic preservation and background diversity. Smaller lower-bound values tend to weaken the representation of tail-class lesions and introduce excessive background interference, whereas larger values reduce the diversity contribution from head-class samples and limit augmentation effectiveness.

The average saliency score of each patch determines its mixing ratio: a higher saliency score indicates a larger probability of the patch belonging to a lesion region, resulting in a larger foreground contribution during feature fusion. In contrast, non-salient patches mainly correspond to background regions and are therefore replaced by backgrounds from head-class samples to increase background diversity while maintaining lesion semantics.

### 3.4. Supervised Contrastive Loss

To further enhance the representation learning of tail-class samples, we employ Supervised Contrastive Learning (SCL) [29], which leverages positive and negative sample sets to encourage the model to learn more discriminative and robust features. Specifically, the positive set for a sample consists of all other samples from the same class, while the negative set includes samples from different classes. SCL contrasts these two sets by minimizing the distance among positive samples and maximizing the distance between positive and negative samples, thereby improving class separability in the feature space. However, when directly applied to long-tailed data, SCL may be biased toward head classes due to their dominance in contrastive pair construction. In our framework, this issue is alleviated by introducing SPMix prior to contrastive learning, which enriches tail-class features and leads to a more balanced feature distribution. Built upon this improved representation space, SCL can more effectively align intra-class features and separate inter-class features, resulting in more stable and discriminative tail-class representations.

During training, we apply SCL to batches of synthetic samples. To ensure each class is equally represented during SCL, we use a class-balanced sampler to select the target-class samples which will be operated upon with the foreground masks, and an instance-balanced sampler to select the contextual samples which will be operated upon with the background masks. Given a synthetic sample $f_{i}$, we define the positive set P(i) as the set of all samples that belong to class *i*. Given a batch of outputs, denoted as *F*, we define the SCL loss function as:

(5) $$L_{i}=\sum_{f_{+}\in P\left(i\right)}\log\frac{\exp(f_{+}\cdot f_{i})}{\sum_{f_{k}\in A\left(i\right)}\exp\left(f_{k}\cdot f_{i}\right)},$$

(6) $$\begin{array}{cc} \; & A\left(i\right)=\left\{f_{k}\in \;\mathrm{queue}\;\cup F\right\}\setminus \left\{f_{k}\in F:k=i\right\},\\ \; & P\left(i\right)=\left\{f_{k}\in A\left(i\right):y_{k}=y_{i}\right\}, \end{array}$$

where A(i) represents the set of all negative and positive samples, excluding $f_{i}$ itself. $y_{i}$ denotes the label of sample $x_{i}$. The queue continuously updates by adding newly encoded representations and removing old ones.

### 3.5. Class Center Rebalance

Inspired by [32,33], we introduce a set of learnable class centers $C=\{c_{1},c_{2},\ldots ,c_{n}\}$ initialized randomly, into the original SCL framework, forming a discriminative semantic feature space where class prototypes act as explicit class-level representations without incurring additional computational cost. By explicitly modeling the relationship between each sample and all class prototypes, the model is encouraged to reason at the class level rather than relying solely on pairwise sample comparisons. This design is particularly beneficial under severe class imbalance, as it enables more stable feature aggregation for underrepresented tail classes. Learnable class centers facilitate tighter intra-class feature clustering while enhancing inter-class separation, leading to more robust and discriminative representations. To further strengthen the role of class centers, we assign them higher weights during training, emphasizing their importance as reference points and mitigating the tendency of class representations to be dominated by majority classes. The loss weight β was empirically set to 0.05 based on preliminary ablation experiments conducted on the validation set. We evaluated several candidate values, including 0.01, 0.05, and 0.15, to balance the contribution between the main classification objective and the auxiliary loss term. Among all evaluated settings, β=0.05 achieved the best balance between overall classification accuracy, F1-score, and long-tailed subset performance. The modified loss function is expressed as:

(7) $$L_{i}=\sum_{f_{+}\in P\left(i\right)\cup c_{y}}-w\left(f_{+}\right)\log\frac{\exp(f_{+}\cdot f_{i})}{\sum_{f_{k}\in A\left(i\right)\cup C}\exp\left(f_{k}\cdot f_{i}\right)},$$

where

(8) $$w\left(f_{+}\right)=\left\{\begin{array}{cc} \beta , & f_{+}\in P\left(i\right)\\ 1.0, & f_{+}\in \left\{c_{y}\right\}. \end{array}\right.$$

Similarity between feature and class centers is first computed, followed by application of the softmax function. The predicted class label is then determined by selecting the class with the highest probability using the argmax function.

## 4. Experiments

### 4.1. Experimental Setup

#### 4.1.1. Datasets

We evaluate TailBoost on three publicly available long-tailed skin cancer datasets with varying class distributions and image characteristics: ISIC 2018 [1], ISIC 2019 [50,51,52], and PAD-UFES-20 [53].

ISIC 2018 is published by the International Skin Imaging Collaboration, a large-scale collection of dermoscopic images designed for skin lesion analysis research. Specifically, it focuses on lesion classification across seven classes: melanocytic nevus (NV), melanoma (MEL), benign keratosis (BKL), basal cell carcinoma (BCC), actinic keratosis (AK), vascular lesion (VASC), and dermatofibroma (DF). The training set contains the following number of samples for each of the seven classes: 6782, 1088, 1075, 483, 289, 99, 70.

ISIC 2019 builds upon the ISIC 2018 collection. The training set comprises the following sample counts across the eight classes: NV (12,875), MEL (4522), BCC (3323), BKL (2624), AK (867), squamous cell carcinoma (SCC) (628), VASC (253), and DF (239). This dataset includes images of varying resolutions, specifically 600 × 450 and 1024 × 1024 pixels.

PAD-UFES-20 is collected in Brazil. It contains 2298 image samples categorized into six classes: BCC, AK, NV, seborrheic keratosis (SEK), SCC, and MEL. Sample distribution across the seven classes in the training set is as follows: 845, 730, 244, 235, 192, 52. The images in this dataset vary in size as they were captured using different smartphone devices, reflecting real-world scenarios where image acquisition conditions are not standardized.

#### 4.1.2. Evaluation Metrics

**Figure 5:**
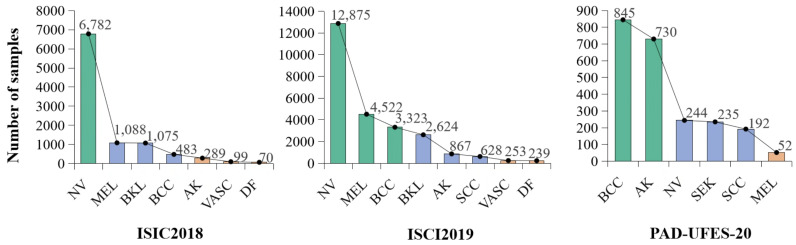
Class distributions of the training sets in our three skin cancer datasets exhibiting long-tailed characteristics. Each dataset is divided into three distinct subsets based on the sample size of each class, represented by different colors: green for “many”, blue for “medium”, and orange for “few”.

The label distributions in these datasets exhibit long-tailed patterns, as illustrated in Figure 5. To evaluate the effectiveness of long-tailed learning methods in addressing the bias introduced by the skewed distribution of the training set, we construct a balanced test set following the experimental setup commonly used for long-tailed problems [30,54]. To enhance the robustness of the evaluation, a larger test set is used compared to our previous work. The sample size for each class in the test set is set to 20% of the number of samples in the class with the fewest instances in the corresponding dataset. This approach ensures a fair assessment of model performance across all classes, particularly those that are underrepresented. To allow a more detailed evaluation and following [23,32,33], we divide each dataset into three subsets: “Many” (with over 3000 images per class), “Medium” (with 300 to 3000 images per class), and “Few” (with fewer than 300 images per class). However, due to the limited sample size of the PAD-UFES-20 dataset, each “Many” class contains more than 600 samples, each “Medium” class contains 60 to 600 samples and each “Few” class contains under 60 samples. We use accuracy as a primary metric to evaluate the performance of each subset, providing insights into the model’s ability to handle classes with varying sample sizes. Additionally, we employ the F1-score, which considers precision and recall, to comprehensively evaluate the model’s classification performance.

#### 4.1.3. Implementation Details

We utilize a Hybrid ViT as the backbone for our experiments, combining a ResNet-50 as the feature extractor with a ViT-S that has a patch size of 16 for further feature processing. The input resolution for all compared methods is set to 224 × 224. All models are trained for 100 epochs with a batch size of 64 on two NVIDIA RTX 3090 GPUs. We employ a cosine annealing schedule. The temperature parameter in the contrastive learning is set to 0.2. RandAug [55] is used for data augmentation. We train the ResNet-50 using the Stochastic Gradient Descent (SGD) optimizer with a momentum of 0.9 and an initial learning rate of 0.025. For the ISIC 2018 and ISIC 2019 datasets, the ViTs are trained with the AdamW optimizer, with a momentum parameter μ=0.9, a weight decay of 0.1, and an initial learning rate of 9 × 10^−5^. For the PAD-UFES-20 dataset, the experimental setup remains consistent with the previous configurations, except for the initial learning rate for the ViTs, which is adjusted to 9× 10^−6^.

The Gaussian filtering and morphological operations in Section 3.2 are only used for saliency map generation and are not applied to the actual training or testing images. All compared methods are trained and evaluated using the original dataset images to ensure a fair comparison. We additionally verified that directly applying hair-removal preprocessing to the input images does not lead to significant performance improvements.

In our experiments, grid search was conducted only on the training set. Specifically, different hyperparameter combinations were explored during training, and the best model checkpoint was selected according to the validation performance (Acc) during training. The final selected model was then evaluated only once on the independent test set. Therefore, there was no direct hyperparameter tuning or model selection performed on the test set. All compared methods are trained under identical settings to ensure a fair comparison, including consistent data splits, a balanced test set, and the same number of training epochs.

### 4.2. Comparisons with State of the Art

We conduct comprehensive comparison experiments with representative state-of-the-art methods employing ResNet-50, ViT-S and Hybrid-ViT as their backbones on three datasets.

#### 4.2.1. ISIC 2018 and ISIC 2019

The BCL, GPaCo, and BPaCo methods employ supervised contrastive learning and are compatible with both ResNet-50 and ViT backbones, whereas other methods are limited to CNN architectures. Our TailBoost adopts Hybrid ViT as its backbone, applying SPMix to features extracted by a CNN.

As tabulated in Table 1, GPaCo improves accuracy for tail classes, but it significantly degrades performance for head classes. In contrast, while CE and BCL perform well in classifying head-class samples, they tend to overlook tail classes. TailBoost not only maintains the high accuracy of head classes but also substantially improves the accuracy for tail classes. Notably, it achieves a significant 5.59% improvement in accuracy and a 5.45% increase in F1-score on the ISIC 2018 dataset, along with a 3.65% improvement in accuracy and a 3.46% boost in F1-score on the ISIC 2019 dataset. The experimental results demonstrate that TailBoost effectively addresses the bias introduced by the long-tailed distribution during training.

#### 4.2.2. PAD-UFES-20

We also tablulate the performance of all compared methods on the PAD-UFES-20 dataset in Table 1. The dataset poses significant challenges due to the extremely limited number of tail-class samples—only 32 samples are available in the training set. This scarcity makes it difficult for most models to effectively learn tail-class features, causing many methods to underperform in recognizing these classes. Despite these challenges, TailBoost provides a balanced solution, achieving the best overall performance across all classes. Specifically, TailBoost outperforms the second-best method, with improvements of 1.67% in accuracy and 0.73% in F1-score. These results demonstrate the effectiveness of TailBoost in addressing the long-tailed problem, particularly in scenarios with extremely limited samples in certain subclasses.

### 4.3. Combining SPMix with Other Methods

SPMix, a mixup-based augmentation strategy, is a key component of TailBoost, which is designed to enhance tail-class representation learning by synthesizing tail-class samples. By leveraging the rich background information from head-class samples to augment tail-class samples—while avoiding the inclusion of head-class lesions—SPMix ensures that the synthetic samples retain semantically consistent labels.

In this section, we explore the role of SPMix as a plug-and-play data augmentation module for other long-tailed learning methods. To evaluate its effectiveness, we integrate SPMix into the data augmentation pipelines of three state-of-the-art contrastive-based methods, including BPaCo, BCL and PaCo. As shown in Table 2, incorporating SPMix consistently leads to performance improvements across all considered frameworks. This integration mitigates the challenges posed by the long-tailed distribution to varying degrees, resulting in superior performance for each method. Notably, the BCL method exhibits a remarkable enhancement of 9.32% in the overall accuracy, with a significant 19.57% increase in the accuracy of the “Few” subset for tail-class samples. These findings underscore the effectiveness of SPMix as a robust data augmentation strategy, demonstrating its ability not only to balance class distributions but also to offer a superior solution for addressing bias.

### 4.4. Ablation Study

In this section, we analyze the impact of several components of TailBoost. All experiments are conducted on the ISIC 2018 dataset. Unless otherwise specified, all settings follow the configuration described in Section 4.1.

#### 4.4.1. Contribution of Each Component

To evaluate the impact of each component in our framework, we conduct ablation studies on the ISIC 2018 dataset. As shown in Table 3, incorporating patch-based mixup to blend head-class and tail-class samples results in notable performance improvements. Refining the patch-based Mixup with saliency guidance yields additional benefits. Class center serves as a key component of the proposed supervised contrastive loss. We show that using either SPMix or class center alone does not improve the overall accuracy. The most significant performance gains are achieved when all modules are integrated, leading to enhancements of 1.24% in accuracy and 1.39% in the F1-score.

#### 4.4.2. Comparision with Other Data Augmentation Methods

We further investigate the contribution of SPMix for TailBoost by substituting it with other augmentation methods including Mixup [10], CutMix [11], SaliencyMix [12] and DiffuseMix [39]. As shown in Table 4, compared with SPMix, other augmentation methods show limited improvements over the baseline. While other augmentation methods generally improve tail-class performance, they achieve it by assigning more synthetic samples to tail classes without adequately embedding the semantic information, such as the diagnostic features. As a result, they may utilize synthetic images with incorrect labels during training, which degrades the representation of head classes and reduces the overall performance. More advanced augmentation strategies like DiffuseMix increasingly rely on diffusion-based generative models, and their effectiveness is largely dependent on the visual quality of the synthesized images. However, in long-tailed medical datasets, tail classes are often underrepresented, with only a limited number of samples available. This scarcity hampers the generative model’s ability to effectively capture the complex and diverse characteristics of tail-class lesions. As a result, the generated images may fail to accurately reflect the true variability of tail-class lesions, leading to unrealistic representations that lack key diagnostic features. These issues may compromise the quality of the synthetic data, hindering the model’s ability to learn meaningful and robust representations for tail classes. Overall, incorporating SPMix into TailBoost yields superior results, outperforming other methods by 2.48% in accuracy and 2.46% in F1-score.

#### 4.4.3. Effect of Different Backbones

To evaluate the impact of backbone combinations on the performance of Hybrid-ViT, we conducted ablation experiments using three distinct backbone pairs: ResNet50 + ViT-S, ResNet101 + ViT-S, ResNet50 + ViT-B, and ResNet101 + ViT-B. The goal was to investigate how the choice of backbone networks influences the model’s ability to capture both fine-grained features and global context. The results of these experiments in Table 5 indicate that while each combination offers unique advantages, the ResNet101 + ViT-B combination achieves the best performance by effectively balancing both fine-grained lesion information and global context.

#### 4.4.4. Computational Complexity and Parameter Comparison

TailBoost achieves competitive performance across all compared methods while maintaining a similar number of parameters and computational cost. As shown in Table 6, TailBoost exhibits nearly identical model complexity to other Hybrid ViT-based methods (e.g., GPaCo and BPaCo), yet consistently outperforms them in terms of both accuracy and F1 scores across all subsets, particularly on the more challenging few-shot tail classes. This demonstrates that the performance improvements of TailBoost are not due to a larger model or higher computational budget, but rather the effectiveness of the proposed saliency-guided mixup and representation rebalancing strategy. Consequently, the enhancements provided by TailBoost are both computationally efficient and impactful for real-world long-tailed medical image classification.

#### 4.4.5. Comparison of Saliency Detection Methods

To investigate the effectiveness of SPMix under different saliency guidance, we conducted experiments using no saliency map, coarse saliency maps, fine-grained saliency maps, and saliency maps generated by SAM [59]. The results show that all forms of saliency guidance lead to clear improvements compared to using no saliency map in Table 7. These results indicate that SPMix can effectively leverage saliency information of varying precision to enhance tail-class feature representation.

#### 4.4.6. Noise for Saliency Maps

We explore the impact of different noise levels ϵ. The addition of random noise is intended to introduce variability, thereby generating diverse outcomes at each iteration. Given that the saliency map is normalized to a range of [−1, 1] during data preparation, we adjust the noise range to align with this interval. After a thorough evaluation, we find that the optimal performance is achieved with a noise range of [−0.5, 0.5], as illustrated in Table 8.

#### 4.4.7. Threshold

Table 9 illustrates the impact of various threshold values on the mixing process. The threshold determines which patches are mixed, those with high saliency scores have mixing ratios of 1. If the threshold is too low, the saliency guidance becomes ineffective, resulting in the loss of lesion regions from the contextual image and the unintended inclusion of lesion regions from the target image. Based on our experimental results, we set the threshold to 0.8, allowing to better represent tail-class features in the synthetic samples.

### 4.5. Visualization

**Figure 6:**
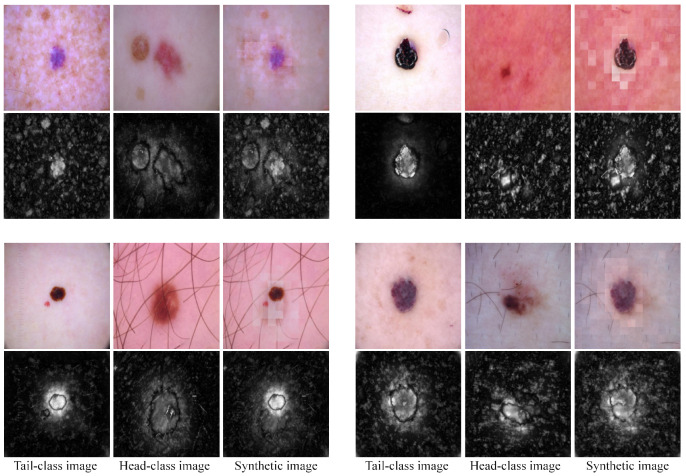
Visualization of synthetic images generated by SPMix at the image level. Four representative cases are displayed. For each case, the second row presents the weighted soft saliency maps corresponding to the images in the first row, where the map in the third column serves as the soft boundary guidance for patch-level fusion.

In this section, we visualize the synthetic images for tail classes generated by SPMix. To better illustrate the functionality of SPMix, we present the results at the image level. As shown in Figure 6, the saliency maps of head-class and tail-class images are first used to compute a weighted foreground map by selecting the maximum saliency response at each spatial location. Unlike binary mask-based augmentation methods, SPMix directly employs this soft saliency map as the blending weight during patch-level synthesis. The weighted saliency map therefore defines the soft transition boundary between foreground lesion regions and background regions. Guided by the weighted foreground map, SPMix preserves diagnostically important lesion structures from tail-class images while introducing diverse background information from head-class samples. This strategy enhances background diversity without disrupting lesion semantics, thereby reducing the risk of introducing incorrect head-class lesion patterns into the synthesized samples.

**Figure 7:**
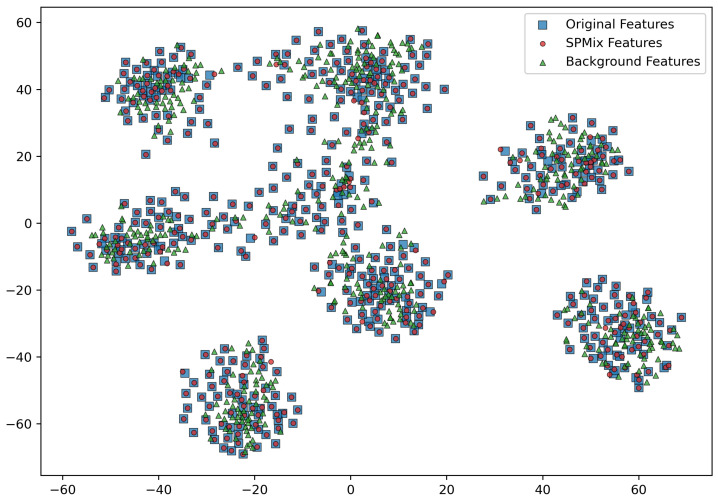
t-SNE visualization of feature distributions for original tail-class samples, SPMix-augmented samples, and background-only mixed samples.

To demonstrate that the proposed SPMix can generate tail-class samples with diverse backgrounds while preserving lesion semantics, we visualize the feature distributions of the original samples, SPMix-augmented samples, and background-only mixed samples using t-SNE. As shown in Figure 7, the feature points corresponding to SPMix-augmented samples almost completely overlap with those of the original samples, forming tightly aligned clusters. This indicates that SPMix preserves the semantic integrity of lesion regions and does not distort the high-level representation of tail-class samples.

## 5. Discussion

Due to the rapid development of deep learning in dermoscopic image analysis, significant progress has been made in skin disease detection, classification, and diagnosis. However, dermoscopic image data often exhibit long-tailed distributions. In scenarios where certain classes are significantly underrepresented, traditional training methods often lead to models that disproportionately favor head classes, resulting in poor generalization to tail classes. Data augmentation, such as Mixup, serves as a crucial motivation for our approach, as it effectively tackles the inherent challenges posed by imbalanced datasets. However, existing mixup methods typically neglect semantic information in the synthetic samples, just simply creating labels based on the proportion of the mixed regions, which may have limited effectiveness in enhancing the learning of discriminative features for tail classes. The key contribution of TailBoost lies in its ability to improve the performance of long-tailed learning methods, particularly through the integration of our pivotal module, SPMix. This module preserves the diagnostic features of tail-class samples using saliency information. It can effectively identify disease-related features, allowing the model to differentiate between lesion areas and backgrounds in tail-class samples. Leveraging the backgrounds of head-class samples to enhance tail-class samples, while preserving critical lesion information through saliency map guidance, SPMix enhances the diversity of tail-class samples through strategic augmentation and tackles the imbalance in class distributions. This approach enables the model to learn more robust and generalizable features across all classes. We demonstrate that this module can be seamlessly integrated with other methods, enhancing their performance and providing a versatile solution for improving long-tailed learning in dermoscopic image analysis.

While TailBoost shows promising results, it has certain limitations. Its dependence on saliency maps, which can be sensitive to noise and other variations, might limit the method’s robustness in more diverse or less controlled settings. Moreover, the effectiveness of SPMix, although demonstrated in this study, may vary when applied to other types of medical images or modalities where the lesion–background contrast is less pronounced. These limitations point to areas for future research, such as enhancing the robustness of saliency map generation, exploring the application of TailBoost across various medical imaging domains, and investigating ways to further simplify and optimize the framework.

## 6. Conclusions

In this study, we extend our previous work, SPMix [14], to develop Tail-synthetic Learning for Boosting Long-tailed Skin Cancer Image Classification (TailBoost). TailBoost enhances performance in long-tailed skin cancer classification. Our approach involves a novel mixup strategy, namely SPMix. SPMix leverages the backgrounds of head-class samples to augment tail-class samples, while preserving crucial tail-class lesion information and minimizing the introduction of head-class lesion features through saliency map guidance. In that case, we construct semantically consistent labels for tail-class samples. We also demonstrate that SPMix can be seamlessly integrated into other long-tailed learning frameworks to boost performance. Given the synthetically balanced dataset, we further enhance tail-class representation learning by incorporating supervised contrastive learning with class center rebalance. Three long-tailed skin cancer classification datasets are adopted to evaluate TailBoost, showing that it outperforms existing state-of-the-art methods.

## Figures and Tables

**Table 1:** Comparison results with state-of-the-art methods on different backbone networks. Test accuracy, Balanced Accuracy (BACC), and F1-score of three datasets are reported. The best results are **bold** while the second best ones are underlined. Statistical significance is determined by a *t*-test. * p<0.005, ** p<0.0005.

Method	Backbone	ISIC2018						ISIC2019						PAD-UFES-20					
		Subsets Acc			Acc	BACC	F1	Subsets Acc			Acc	BACC	F1	Subsets Acc			Acc	BACC	F1
		Many	Med	Few				Many	Med	Few				Many	Med	Few			
CE [19]	ResNet-50	**97.83**	51.45	36.23	51.55	61.84	47.98	69.10	27.08	40.10	46.09	45.43	44.10	82.50	36.67	0.00	45.83	39.72	36.48
CE-resample [21]		84.78	62.32	52.17	61.18	66.42	60.12	59.72	53.68	67.19	51.82	60.20	51.31	72.50	48.33	20.00	51.67	46.94	49.43
ResLT [56]		73.91	21.74	0.00	19.88	31.88	12.44	80.90	6.25	63.02	48.44	50.06	42.16	57.50	6.67	5.00	23.33	23.06	16.62
BCL [33]		76.09	53.62	73.19	65.22	67.63	65.19	62.50	42.01	78.12	58.72	60.88	57.74	77.50	35.00	20.00	46.67	44.17	42.01
GPaCo [57]		33.33	50.00	**87.68**	59.01	57.00	55.45	40.97	48.61	**93.75**	57.03	61.11	55.85	17.50	38.33	**90.00**	40.00	48.61	32.98
MDCS [58]		82.61	73.19	66.67	71.71	74.16	71.90	66.67	57.99	81.25	67.06	68.64	66.62	70.00	48.33	20.00	50.83	46.11	48.30
BPaCo [54]		95.65	60.14	41.30	57.14	65.70	54.72	79.17	39.58	74.48	63.15	64.41	62.11	72.50	35.00	0.00	41.67	35.83	33.73
ConCutmix [38]		82.61	56.52	76.09	73.70	71.74	68.26	55.21	46.18	87.50	59.90	62.96	58.61	47.50	45.00	55.00	47.50	49.17	45.46
ProCo [34]		86.96	70.29	76.81	75.47	78.02	75.81	78.82	71.53	85.42	77.73	78.59	77.62	75.00	45.00	35.00	53.33	51.67	49.85
CE [19]	ViT-S	91.30	78.99	75.36	79.19	81.88	78.63	87.15	68.40	76.56	77.47	77.37	77.52	**85.00**	53.33	25.00	59.17	54.44	52.31
CE-resample [21]		84.78	76.81	79.71	79.19	80.43	79.24	79.17	69.79	84.90	77.08	77.95	77.37	70.00	61.67	25.00	58.33	52.22	57.07
BCL [33]		91.30	78.26	60.87	72.67	76.81	72.60	64.58	33.68	62.50	52.47	53.59	49.79	67.50	53.33	40.00	55.83	53.61	54.76
GPaCo [57]		43.48	**83.70**	**87.68**	73.91	71.62	69.18	71.18	**82.64**	89.58	80.08	81.13	79.84	32.50	**76.67**	70.00	60.83	59.72	56.19
BPaCo [54]		91.30	74.64	65.94	73.29	77.29	73.07	**89.93**	75.00	80.73	82.03	81.89	82.12	82.50	51.67	10.00	55.00	48.06	48.61
CE [19]	Hybrid ViT-S	95.65	79.71	68.12	77.02	81.16	77.20	84.38	68.06	71.88	75.13	74.77	74.99	82.50	48.33	20.00	55.00	50.28	51.82
CE-resample [21]		95.65	73.91	70.29	75.47	79.95	75.93	85.76	64.24	77.60	75.65	75.87	76.09	70.00	63.33	25.00	59.17	52.78	57.80
BCL [33]		93.48	72.46	63.04	71.43	76.33	71.38	60.07	43.06	56.25	52.73	53.13	51.23	52.50	50.00	40.00	49.17	47.50	48.45
GPaCo [57]		60.87	72.46	85.51	76.40	72.95	75.76	73.26	81.60	92.19	81.12	82.35	81.17	35.00	63.33	80.00	56.67	59.44	51.82
BPaCo [54]		91.30	72.46	78.26	77.64	80.67	77.93	88.19	77.43	80.21	82.16	81.94	82.45	82.50	46.67	25.00	55.00	51.39	50.42
Ours		89.13	80.43	**87.68**	84.78 **	**85.75** **	**84.69** **	88.19	81.25	89.06	**85.81** **	**86.17** **	**85.91** **	35.00	73.33	85.00	**62.50** *	**64.44** *	**58.53** *

**Table 2:** Performance of different state-of-the-art frameworks with SPMix on the ISIC 2018 dataset. The better ones are **bold**.

Method	Subsets Acc			Acc	F1-Score
	Many	Med	Few		
BPaCo [54]	91.30	72.46	**78.26**	77.64	77.93
BPaCo + SPMix	**93.48**	**79.71**	73.91	**79.19**	**79.33**
BCL [33]	**93.48**	72.46	63.04	71.43	71.38
BCL + SPMix	91.30	**75.36**	**82.61**	**80.75**	**81.10**
PaCo [32]	67.39	73.19	**88.41**	78.88	78.68
PaCo + SPMix	**82.61**	**76.09**	87.68	**81.99**	**82.30**

**Table 3:** Impact of each component on TailBoost’s performance. The best ones are **bolded**.

Patch-Based Mixup	Saliency Guidance	Class Center	Subsets Acc			Acc	F1-Score
			Many	Med	Few		
✗	✗	✓	60.87	72.46	85.51	76.40	75.76
✓	✗	✓	67.39	76.81	**89.86**	81.06	80.94
✗	✓	✓	80.41	79.71	88.41	83.54	83.30
✓	✓	✗	**93.48**	76.09	68.12	75.16	75.16
✓	✓	✓	89.13	**80.43**	87.68	**84.78**	**84.69**

**Table 4:** Performance of TailBoost with different data augmentation strategies. The best ones are **bolded**.

Method	Subset Acc			Acc	F1-Score
	Many	Med	Few		
TailBoost with Mixup [10]	39.13	76.09	**91.30**	77.33	76.48
TailBoost with CutMix [11]	76.09	69.57	87.68	78.26	78.36
TailBoost with DiffuseMix [39]	86.96	87.68	69.57	79.81	79.69
TailBoost with SaliencyMix [12]	71.74	78.26	89.86	82.30	82.23
TailBoost with SPMix (Proposed)	**89.13**	**80.43**	87.68	**84.78**	**84.69**

**Table 5:** Performance of TailBoost with different backbones. The best ones are **bolded**.

Backbone	Many	Med	Few	Acc	F1
ResNet50 + ViT-S	89.13	80.43	**87.68**	84.78	84.69
ResNet101 + ViT-S	89.13	84.06	84.06	84.78	85.02
ResNet50 + ViT-B	86.96	84.06	85.51	85.09	85.51
ResNet101 + ViT-B	**91.30**	**86.96**	82.61	**85.71**	**85.68**

**Table 6:** Computational complexity and parameter comparison between TailBoost and other long-tailed learning methods.

Method	Backbone	Number of Parameters (M)	FLOPs (GFLOPs)
ProCo	ResNet-50	29.807	4.138
BCL	Hybrid ViT-S	59.567	13.034
GPaCo	Hybrid ViT-S	56.715	13.023
BPaCo	Hybrid ViT-S	56,617	13.023
TailBoost (ours)	Hybrid ViT-S	56.616	13.023

**Table 7:** Effect of saliency map quality on classification performance. The best ones are **bolded**.

Method	Subset Acc			Acc	F1-Score
	Many	Med	Few		
Without saliency map	67.39	76.81	**89.86**	81.06	80.94
Saliency map generated by SAM	82.61	80.43	86.96	83.54	83.51
Coarse saliency map	86.96	**82.62**	85.51	84.47	84.43
Fine-grained saliency map	**89.13**	80.43	87.68	**84.78**	**84.69**

**Table 8:** Performance of TailBoost with different ranges of Noise. The best ones are **bolded**.

Noise	Subsets Acc			Acc	F1-Score
	Many	Med	Few		
[−1, 1]	**89.13**	**84.06**	82.61	84.16	84.19
[−0.5, 0.5]	**89.13**	80.43	**87.68**	**84.78**	**84.69**
[−0.1, 0.1]	84.78	77.54	85.51	81.99	82.26

**Table 9:** Performance of TailBoost with different values of threshold. The best ones are **bolded**.

Threshold	Subsets Acc			Acc	F1-Score
	Many	Med	Few		
1	**91.30**	76.09	**89.86**	84.16	84.13
0.9	**91.30**	**83.33**	83.33	84.47	84.31
0.8	89.13	80.43	87.68	**84.78**	**84.69**
0.7	76.09	77.54	**89.86**	82.61	82.47
0.6	86.96	78.26	88.41	83.85	83.99

## Data Availability

The publicly available datasets used in this study include ISIC2018, ISIC2019 and PAD-UFES-20. All datasets can be accessed from their official published repositories.
